# Urinary long non-coding RNA GAS5 as a noninvasive diagnostic biomarker for renal fibrosis

**DOI:** 10.1080/0886022X.2025.2534493

**Published:** 2025-07-20

**Authors:** Ying Yu, Yang-yang Niu, Ying-ying Zhang, Chen Yu

**Affiliations:** Department of Nephrology, School of Medicine, Tongji Hospital, Tongji University, Shanghai, China

**Keywords:** GAS5, biomarker, renal fibrosis, serum, urine

## Abstract

**Background:**

Renal fibrosis, the terminal pathological pathway of chronic kidney disease (CKD), lacks reliable noninvasive biomarkers for clinical assessment. Current diagnostic reliance on renal biopsy—despite its gold-standard status—poses risks of bleeding and sampling errors, necessitating alternatives. Long non-coding RNA growth arrest-specific 5 (GAS5) regulates fibrogenic pathways, but its utility as a liquid biopsy marker remains unexplored.

**Method:**

This prospective cross-sectional study quantified GAS5 levels in paired plasma and urine samples from 198 CKD patients (stratified by renal fibrosis scoring: mild: <25%, moderate: 25–50%, severe: ≥50% fibrotic area) and 20 healthy controls. Multivariate regression models adjusted for cardiorenal confounders (hypertension, blood pressure, and so on) evaluated GAS5’s association with fibrosis.

**Results:**

Plasma GAS5 levels increased with renal fibrosis severity, whereas urinary GAS5 exhibited progressive suppression. After adjusting for factors such as hypertension history, systolic and diastolic blood pressure, blood urea nitrogen, creatinine, eGFR, and uric acid, use of ACEI/ARB, multivariate analysis showed significant associations between urinary GAS5 level and renal fibrosis. ROC analysis revealed urinary GAS5’s superior diagnostic accuracy compared with eGFR and TGF-β1. AUCs of urinary GAS5 were even higher in moderate and severe renal fibrosis group.

**Conclusions:**

While plasma GAS5 upregulation and urinary GAS5 suppression reflect inverse expression patterns in renal fibrosis, urinary GAS5 emerges as the dominant noninvasive biomarker due to its superior diagnostic performance (AUC = 0.868) and clinical accessibility.

## Graphical Abstract

**Figure:**
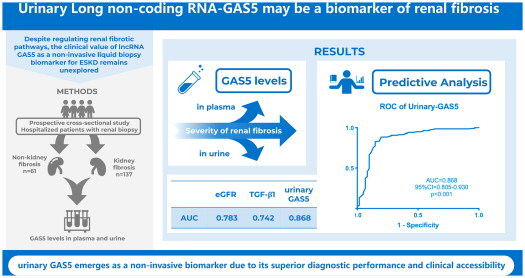

## Introduction

Chronic kidney disease (CKD), affecting over 13% of the global population, is characterized by progressive renal function decline and fibrosis, the latter being the primary driver of disease progression to end-stage renal disease (ESRD) [1–5]. Despite advancements in treatment, current diagnostic tools remain inadequate. While renal biopsy remains the gold standard for assessing fibrosis, its invasive nature and associated risks (e.g. bleeding, sampling errors) limit clinical utility, particularly for repeated monitoring. Conventional biomarkers like serum creatinine and eGFR lack sensitivity to early fibrotic changes, and imaging modalities (e.g. ultrasound) offer limited specificity [6–8]. This diagnostic gap underscores the urgent need for noninvasive biomarkers capable of detecting and quantifying renal fibrosis.

Long non-coding RNAs (lncRNAs), a class of regulatory RNAs (>200 nucleotides), modulate gene expression through epigenetic, transcriptional, and post-transcriptional mechanisms, playing pivotal roles in fibrotic diseases [9,10]. Originally identified in growth-arrested cells, GAS5 regulates fibrotic cascades across multiple organ systems by modulating cell proliferation and apoptosis through key signaling molecule interactions [11–13]. GAS5 suppresses fibrogenesis by sponging pro-fibrotic microRNAs (e.g. miR-21) [14] and modulating downstream pathways such as TGF-β/Smad3 [15,16]. Preclinical studies demonstrate that GAS5 expression is downregulated in renal fibrosis models, including unilateral ureteral obstruction (UUO) and diabetic nephropathy, correlating with extracellular matrix (ECM) accumulation. In humans, reduced urinary GAS5 levels associate with advanced fibrosis in small CKD cohorts [14].

While noninvasive biomarkers for renal fibrosis, such as KIM-1 and EGF, lack specificity or predictive utility for disease progression [7,8], GAS5 emerges as a promising candidate due to its dual regulatory roles in suppressing fibroblast activation and enhancing tubular repair, addressing a critical gap in current clinical practice. This prospective cohort study aims to quantify GAS5 expression across CKD fibrosis stages, compare its diagnostic accuracy with established markers (e.g. eGFR, TGF-β1) and imaging-based fibrosis scores, and elucidate the mechanistic basis of its dual-tissue regulation (plasma/urine dynamics), thereby bridging molecular insights with clinical validation to establish GAS5 as a novel, accessible biomarker for fibrosis and redefine management paradigms in CKD.

## Materials and methods

### Patients and tissue specimens

Between October 2017 and December 2020, 384 patients who underwent renal biopsy in the Nephrology Department of Shanghai Tongji Hospital were initially involved. After excluding those with incomplete baseline data, missing blood or urine samples, acute pathological changes, 198 patients remained for analysis. General clinical data (age, sex, history of hypertension/diabetes/coronary heart disease/cerebral stroke/tumor, administion of diuretics/CCB/ACEI/ARB within 1 week before specimen collection, systolic and diastolic blood pressure), laboratory results (blood urea nitrogen, creatinine, uric acid, eGFR, albumin, and 24-h urine protein), and plasma and urine samples were collected for each patient one day before kidney biopsy. Additionally, plasma and urine samples were collected from 20 healthy controls, matched by age and sex. The study protocol was approved by the Ethics Committee of Shanghai Tongji Hospital (reference number: KYSB-2019-008). All patients signed an informed consent before enrollment. All renal biopsy specimens were subjected to Masson’s staining, and two renal pathologists independently performed fibrosis scoring using ImageJ software.

### Elisa

The levels of TGF-β1 and IL-6 in plasma were detected by specific Quantikine sandwich ELISA kits for human TGF-β1 (R & D Systems, # DY240) and IL-6 (R & D Systems, # D6050B).

### Sample processing

Blood (EDTA tubes) was processed within 1 h using a two-step centrifugation (4 °C): 2000 × g for 10 min to remove cellular debris, followed by 12,000 ×g for 10 min to isolate plasma. Urine was centrifuged (12,000 ×g, 4 °C, 10 min), and supernatants stored at −80 °C.

### Plasma and urine GAS5 detection

RNA was extracted from plasma or urine samples using the RNAiso Blood kit (Takara 9112): 250 μL plasma/urine + 750 μL reagent, incubated at RT for 5 minutesChloroform (200 μL) was added, vortexed, and centrifuged (12,000 ×g, 4 °C, 15 min); RNA supernatant was mixed with isopropanol (equal volume), incubated (10 min, RT), and centrifuged (12,000 ×g, 4 °C, 10 min); Pellets were washed with 75% ethanol (7,500 ×g, 4 °C, 5 min), air-dried, and dissolved in RNase-free water. Then it was followed by reverse transcription using the PrimeScript^™^ RT reagent Kit with gDNA Eraser (Takara RR047). Real-time PCR was used to measure GAS5 expression levels in plasma or urine samples. The level of urinary creatinine was measured in the clinical laboratory of Tongji Hospital in Shanghai. The measured urine GAS5 result was divided by urine creatinine ×1000 to obtain the standardized urine GAS5 result, which was used as the final urinary GAS5. Primers used included human-GAS5-F (CTTCTGGGCTCAAGTGATCCT), human-GAS5-R (TTGTGCCATGAGACTCCATCAG), human-GAPDH-F (GCACCGTCAAGGCTGAGAAC), and human-GAPDH-R (TGGTGAAGACGCCAGTGGA).

### Statistical analysis

All continuous data are presented as mean ± standard deviation. Categorical variables are displayed as frequencies and percentages. ANOVA/Mann-Whitney was used to assess differences in GAS5 expression among groups, with the LSD test employed for pairwise comparisons. Spearman’s correlation coefficient was used to evaluate the correlation between two variables. A binary logistic regression model was applied to analyze risk factors associated with renal fibrosis. The predictive ability of different indicators for renal fibrosis was determined using receiver operating characteristic (ROC) curve analysis. Optimal cutoff values were selected by maximizing Youden’s index (sensitivity + specificity −1) to balance diagnostic accuracy, with sensitivity, specificity, and area under the curve (AUC) being examined. A *p*-value of < 0.05 was considered statistically significant. Statistical analyses were conducted using SPSS 16.0 (SPSS Inc., Chicago, IL, USA).

## Results

### Clinical characteristics

**Figure 1.**
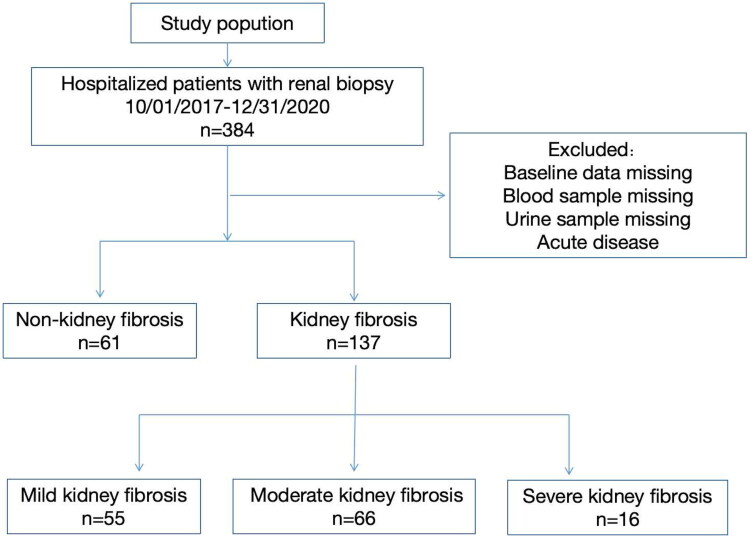
Flowchart of clinical specimen.

The investigation enrolled 198 consecutive patients undergoing diagnostic renal biopsy, with cohort stratification guided by quantitative histopathological assessment of fibrosis severity. The study population comprised 61 non-fibrotic controls (30.8%) and 137 fibrotic cases (69.2%), subsequently categorized into three pathological subgroups: mild fibrosis (fibrosis score <25%, *n* = 55, 40.1%), moderate fibrosis (fibrosis score: 25–50%, *n* = 66, 48.2%), and severe fibrosis (fibrosis score ≥ 50%, *n* = 16, 11.7%) (Figure 1). A supplementary cohort of 20 age-matched healthy volunteers provided baseline biological specimens (plasma/urine) for comparative analyses.

### Comparative analysis of clinical parameters

Demographic and biochemical profiling revealed no statistically significant intergroup disparities between the fibrotic and non-fibrotic cohorts regarding sex distribution, age, serum albumin concentrations, 24-h urinary protein excretion, history of coronary heart disease/cerebral stroke/tumor, administion of diuretics, or IL-6 (all *p* > 0.05). In contrast, marked clinical divergences were observed in cardiometabolic parameters: the fibrotic cohort exhibited significantly higher prevalence rates of diabetes mellitus (DM) and hypertension (*p* < 0.001), elevated systolic/diastolic blood pressure, administion of CCB/ACEI/ARB (*p* < 0.05), and exacerbated renal dysfunction biomarkers including blood urea nitrogen (BUN), serum creatinine, uric acid, TGF-β1 and reduced estimated glomerular filtration rate (eGFR) (*p* < 0.001 for all) (Table 1).

**Table 1.** Baseline clinical characteristics of patients undergoing renal biopsy.

	NF(*n* = 61)	F (*n* = 137)	*p*-value
Male, *n*(%)	40 (65.57)	81(59.12)	0.393
Age, years	48.38 ± 14.40	52.23 ± 16.28	0.113
DM, *n*(%)	2 (3.28)	74 (54.01)	0.000
HBP, *n*(%)	11 (18.03)	92(67.15)	0.000
CHD, *n*(%)	1 (1.64)	8 (5.84)	0.191
Stroke, *n*(%)	0 (0)	8 (5.84)	0.055
Tumor, *n*(%)	0 (0)	5 (3.65)	0.132
Diuretics, *n*(%)	2 (3.28)	11 (8.03)	0.214
CCB, *n*(%)	4 (6.56)	40 (29.20)	0.000
ACEI, *n*(%)	4 (6.56)	24 (17.52)	0.042
ARB, *n*(%)	4 (6.56)	24 (17.52)	0.042
SBP, mmHg	126.10 ± 13.47	145.19 ± 22.29	0.000
DBP, mmHg	80.36 ± 11.40	85.64 ± 11.54	0.003
ALB, g/L	28.59 ± 10.84	31.42 ± 8.83	0.076
BUN, mmol/L	6.17 ± 2.88	9.38 ± 6.43	0.000
Scr, μmol/L	84.77 ± 26.09	155.53 ± 115.20	0.000
eGFR, mL/min/1.73 m^2^	88.29 ± 22.00	58.43 ± 30.68	0.000
UA, μmol/L	373.67 ± 101.39	408.10 ± 115.02	0.045
UPRO, g/24h	3.88 ± 3.94	5.06 ± 4.64	0.086
IL-6, pg/mL	48.86 ± 8.61	111.8404 ± 37.68	0.097
TGF-β1, pg/mL	103.64 ± 11.93	231.61 ± 22.80	0.000

NF, non-fibrosis group; F, renal fibrosis group; Male, gender; Age, age; DM, diabetes history; HBP, hypertension history; CHD, Coronary Heart Disease history; Stroke, Cerebral Stroke history; Tumor, tumor history; Diuretics/CCB/ACEI/ARB, Medications were administered within 1 week before specimen collection; SBP, systolic blood pressure; DBP, diastolic blood pressure; ALB, albumin; BUN, blood urea nitrogen; Scr, serum creatinine; eGFR, estimated glomerular filtration rate; UA, uric acid; UPRO, 24-h urine protein; IL-6, Interleukin-6; TGF-β1, Transforming Growth Factor-beta 1.

### Histopathological spectrum across study cohorts

**Figure 2.**
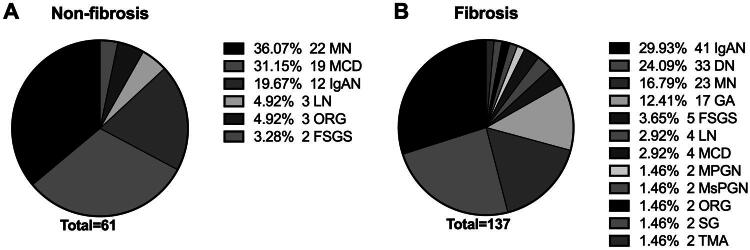
Pathological diagnoses of renal biopsy patients. IgAN, IgA nephropathy; DN, diabetic nephropathy; MN, membranous nephropathy; GA, glomerular arteriosclerosis; FSGS, focal segmental glomerulosclerosis; LN, lupus nephritis; MCD, minimal change disease, including mild kidney disease; MGPN, membranoproliferative glomerulonephritis; MsGPN, mesangial proliferative glomerulonephritis; ORG, obesity-related nephropathy; SG, glomerulosclerosis; TMA, thrombotic microangiopathy.

The non-fibrotic cohort exhibited a histopathological profile dominated by membranous nephropathy (MN), IgA nephropathy (IgAN), minimal change disease (MCD), and mild renal parenchymal injury. Conversely, the fibrotic cohort demonstrated distinct disease distribution patterns, with diabetic nephropathy (DN) emerging as the predominant etiology, followed by IgAN and MN, collectively constituting the primary diagnostic entities (Figure 2).

### Plasma GAS5 expression levels in renal fibrosis cohorts

**Figure 3.**
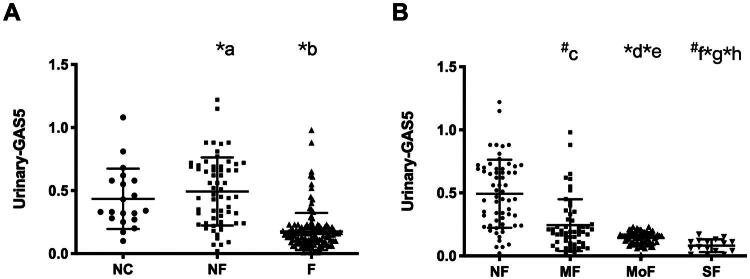
GAS5 Expression levels in plasma. (A) Comparison between healthy controls (NC), non-fibrosis (NF), and fibrosis groups (F). (B) Stratified analysis by fibrosis severity: mild (MF), moderate (MoF), severe (SF). *p < 0.001, ^#^*p* < 0.05. a: NF *vs.* NC, b: F *vs.* NC, c: MF *vs.* NF, d: MoF *vs.* NF, e: MoF *vs.* MF, f: SF *vs.* NF.

Quantitative analysis revealed a statistically significant elevation in plasma GAS5 expression levels within the renal fibrosis cohort compared to both healthy controls (*p* < 0.001) and non-fibrotic pathological controls (*p* < 0.001). Notably, no significant intergroup difference was observed between non-fibrotic subjects and healthy controls (Figure 3(A)). Stratified analysis demonstrated progressive upregulation of plasma GAS5 expression levels corresponding to fibrosis severity. The mild fibrosis subgroup exhibited markedly elevated GAS5 expression compared to non-fibrotic controls (*p* = 0.014), with subsequent significant increments observed in moderate (vs. mild, *p* < 0.001). Although the severe fibrosis cohort showed numerically higher GAS5 levels relative to both mild and moderate subgroups, these inter-group differences did not reach statistical significance (Figure 3(B)).

### Urinary GAS5 expression levels

**Figure 4.**
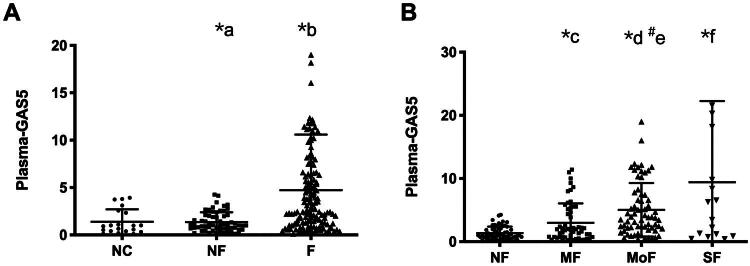
Urinary GAS5 expression levels. (A) Urinary GAS5 expression levels in healthy control people and in patients with or without renal fibrosis. (B) Urinary GAS5 expression in patients with different degrees of renal fibrosis. *p < 0.001, ^#^*p* < 0.05. a: NF *vs.* NC, b: F *vs.* NC, c: MF *vs.* NF, d: MoF *vs.* NF, e: MoF *vs.* MF, f: SF *vs.* NF, g: SF *vs.* MF, h: SF *vs.* MoF.

Urinary GAS5 expression demonstrated significant differential expression across study cohorts, as illustrated in Figure 4. Quantitative analysis revealed markedly decreased GAS5 expression levels in the renal fibrosis group compared with both healthy controls (*p* < 0.001) and non-fibrosis pathological counterparts (*p* < 0.001) (Figure 4(A)). Subsequent stratification by histopathological severity (Figure 4(B)) revealed a graded reduction in urinary GAS5 levels corresponding to advancing fibrotic stages. Specifically, cohorts with mild, moderate, and severe fibrosis exhibited progressively diminishing GAS5 expression relative to non-fibrotic controls (all intergroup comparisons *p* < 0.001). Importantly, pairwise analyses confirmed statistically significant differences between all adjacent fibrotic stages (mild vs moderate: *p* = 0.003; moderate vs severe: *p* < 0.001), establishing a robust inverse correlation between urinary GAS5 levels and histological progression of renal fibrosis.

### Correlation analysis of plasma or urinary GAS5 with renal fibrosis

**Figure 5.**
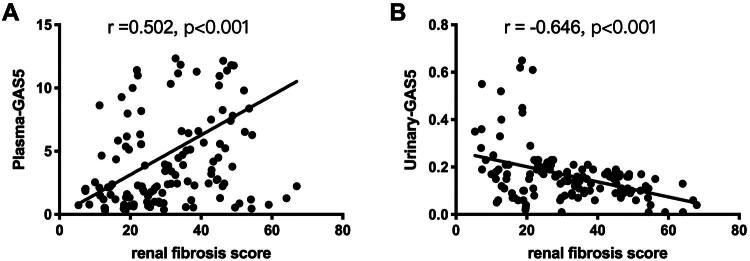
Correlation analysis of plasma GAS5 and urinary GAS5 with renal fibrosis. (A) Correlation between plasma GAS5 levels and the areas of renal fibrosis. (B) Correlation between urinary GAS5 levels and the areas of renal fibrosis.

Following normality testing, the expression levels of GAS5 in both plasma and urine were found to be non-normally distributed. Spearman’s rank correlation analysis revealed a robust positive association between plasma GAS5 and renal fibrosis severity (*r* = 0.502, 95% CI 0.43–0.58, *p* < 0.001; Figure 5(A)), whereas urinary GAS5 exhibited a stronger inverse correlation (*r* = −0.646, 95% CI −0.71–−0.58, *p* < 0.001; Figure 5(B)). Fisher’s z-transformation confirmed the magnitude difference between correlations (|z| = 3.12, *p* = 0.002), underscoring urinary GAS5’s superior discriminative capacity for fibrotic progression (Figure 5).

### Multivariable logistic regression modeling

A binary logistic regression model was constructed, incorporating covariates demonstrating univariate significance (*p* < 0.05 for hypertension, diabetes mellitus, systolic blood pressure, diastolic blood pressure, blood urea nitrogen, serum creatinine, eGFR, uric acid, CCB, ACEI, ARB, plasma GAS5, and urinary GAS5). The analysis revealed that urinary GAS5, eGFR and hypertension/diabetes history had *p*-value less than 0.05, indicating a statistically significant association with the occurrence of renal fibrosis after adjusting for confounding factors. Among these factors, urinary GAS5 had the highest correlation coefficient, suggesting a potentially greater impact on renal fibrosis, indicating that urinary GAS5 may serve as a potential biomarker for renal fibrosis (Table 2). The interaction effect test showed that the interaction between urinary GAS5 and hypertension, urinary GAS5 and diabetes was not significant (*p* = 0.365, 0.629) (Table 3). A formula for predicting renal fibrosis was obtained: *P*(*Y* = 1) = 1/(1 + e^(−1.461 – 3.445 × DM + 6.588 × UGAS5–2.135 × HBP)).

**Table 2.** Risk factors for renal fibrosis.

Variable	β	OR	95% CI	*p* value
HBP	1.390	42.556	(4.753–381.029)	0.001
DM	3.751	4.014	(1.414–11.396)	0.009
eGFR	−0.022	.979	(0.961–0.996)	0.017
U-GAS5	−6.353	.002	(0.000–0.021)	0.000

DM, diabetes history; HBP, hypertension history; eGFR, estimated glomerular filtration rate; U-GAS5, urinary GAS5.

**Table 3.** Risk factors for renal fibrosis (effect of interaction).

Variable	*β*	OR	95% CI	*p* value
DM	3.445	31.35	0.75–1308.5	0.070
U-GAS5	−6.588	0.001	0.00–0.03	0.000
HBP	2.135	8.46	1.43–50.0	0.019
U-GAS5 ×HBP	−2.294	0.10	0.00–14.4	0.365
U-GAS5 ×DM	1.439	4.21	0.01–1436.5	0.629

DM, diabetes history; HBP, hypertension history; U-GAS5, urinary GAS5.

### Predictive values of plasma GAS5 and urinary GAS5 for renal fibrosis

**Figure 6.**
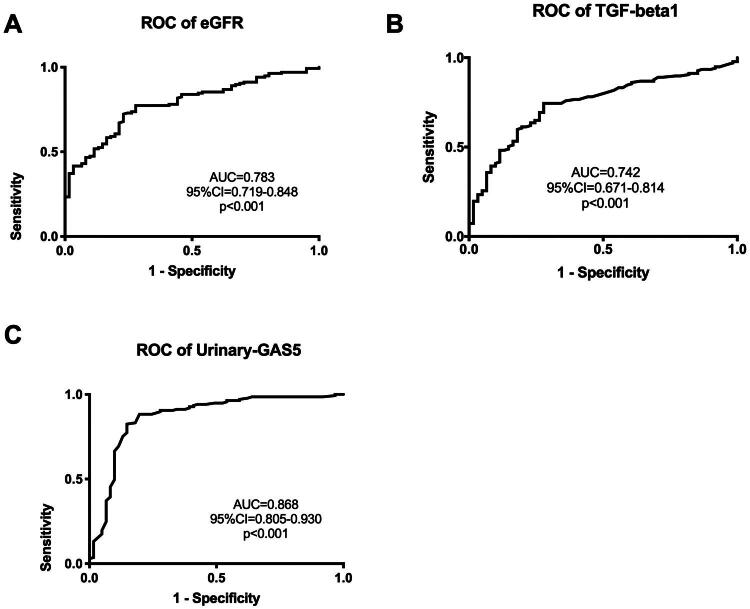
Predictive analysis of various indicators for renal fibrosis. ROC curves for prediction of renal fibrosis according to eGFR (A), TGF-β1 (B) and urinary GAS5 level (C) of patients.

**Figure 7.**
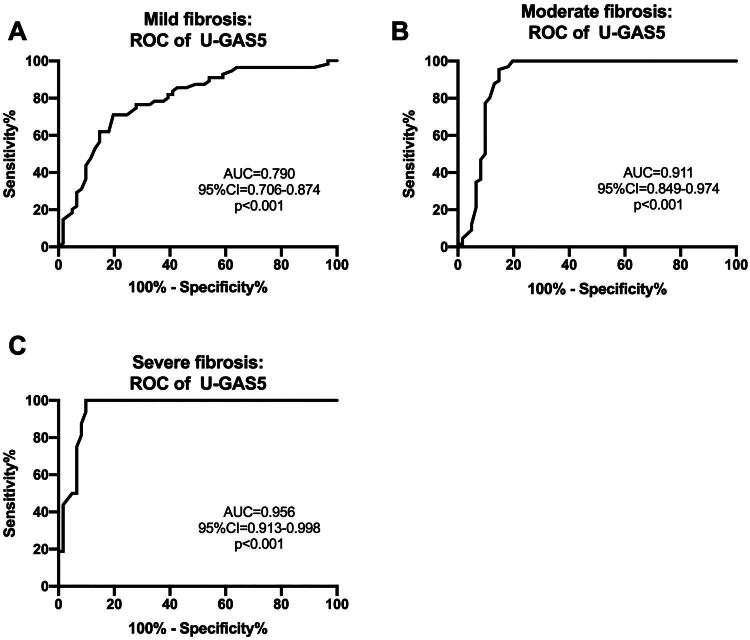
Predictive analysis of urinary GAS5 for renal fibrosis: Stratified analysis by severity. (A) Mild fibrosis group; (B) Moderate fibrosis group; (C) Severe fibrosis group.

Further analysis was conducted using ROC curves to evaluate the predictive value of GAS5 for renal fibrosis. As shown in Figure 6(A) and (B), the area under the curve (AUC) of eGFR and TGF-β1 in predicting renal fibrosis were 0.783 (95% confidence interval [CI], 0.719–0.848) and 0.742 (95% CI, 0.671–0.814) respectively, whereas, the AUC of urinary GAS5 was 0.868 (95% CI, 0.805–0.930) (Figure 6(C)). The results showed that, compared to eGFR and TGF-β1, urinary GAS5 had a higher AUC, suggesting that urinary GAS5 has a superior predictive capability for renal fibrosis. The cutoff value for urinary GAS5 was 0.235 (sensitivity 88.32%, specificity 80.33%), which could serve as a noninvasive diagnostic threshold to distinguish patients with renal fibrosis from non-fibrotic individuals in clinical practice. Furthermore, the AUC of urinary GAS5 was also high in the three renal fibrosis subgroups (Figure 7), especially higher in the more severe renal fibrosis group. In the severe renal fibrosis subgroup, the AUC value of urinary GAS5 was as high as 0.956.

## Discussion

The progression of CKD to end-stage renal disease is universally characterized by glomerular sclerosis and increased extracellular matrix deposition in the renal interstitium, also known as renal fibrosis [17,18]. Early diagnosis and targeted intervention at the onset of renal fibrosis are clinically significant.

In clinical practice, traditional indicators such as proteinuria and serum creatinine levels are commonly employed to assess the progression of CKD and infer renal fibrosis [5,19]. However, due to the kidney’s extensive compensatory capacity, these markers often only become prominent when renal lesions are already severe, typically indicating moderate to advanced stages of renal fibrosis. This lag in biomarker responsiveness underscores a critical gap in early detection and monitoring.

Moreover, renal biopsy, the current gold standard for measuring renal fibrosis, is an invasive procedure with inherent risks such as hemorrhage and infection [20,21]. These associated risks limit its feasibility for repeated assessments, underscoring the critical need for noninvasive, repeatable biomarkers that can reliably detect renal fibrosis at earlier stages.

Circulating lncRNAs are typically regarded as unstable entities due to the heightened activity of ribonucleases (RNases) in plasma, which possess the capability to degrade a myriad of RNA species, including lncRNAs. Indeed, elevated levels of plasma RNases have been documented in cancer patients [22], posing a significant challenge. However, an array of studies has demonstrated that circulating lncRNAs exhibit remarkable resilience, maintaining their integrity even when subjected to direct digestion by RNase A or exposed to other harsh conditions [23,24]. Furthermore, it has been elucidated that lncRNA expression profiles can be discerned in the bodily fluids of patients afflicted with cancer or cardiovascular diseases [25,26], providing crucial diagnostic insights.

Of particular note, lncRNA-TapSAKI has been observed at elevated concentrations in the plasma of individuals suffering from acute kidney injury relative to healthy controls and has been recognized as an independent prognostic indicator of 28-day mortality in these patients [27]. Our research group has successfully quantified GAS5 expression in both the plasma and urine of patients spanning various stages of CKD [14]. Nonetheless, prior investigations were constrained by limited sample sizes and concentrated exclusively on the correlation between GAS5 levels in plasma and urine and the progression of CKD, neglecting to explore its potential association with the severity of renal fibrosis in these patients.

The present study demonstrates that the expression of plasma GAS5 and urinary GAS5 in renal fibrosis were in opposite trends: plasma GAS5 upregulation and urinary GAS5 suppression. These findings suggest that during renal fibrosis, GAS5 expression is suppressed within renal tissue, leading to reduced deposition in tissue; simultaneously, due to impaired renal function, the excretion of GAS5 in urine decreases, resulting in elevated plasma GAS5 levels. This reciprocal relationship may be mechanistically linked to: (1) Stress-induced extrusion of cellular RNA contents into circulation *via* extracellular vesicles, as demonstrated in podocyte injury models [28]; (2) Impaired tubular reabsorption mechanisms mediating urinary RNA loss, consistent with observations in diabetic nephropathy [29].

The specific mechanisms underlying GAS5 expression changes in fibrosis remain unclear. Our prior studies [14,16] have demonstrated that GAS5 inhibits renal fibrosis *via* microRNA-mediated pathways, including suppression of miR-21 activity and regulation of the Smad3/miRNA-142-5p axis in TGF-β signaling. The observed plasma-urine concentration gradient aligns with the emerging paradigm of circulating lncRNAs as damage-associated molecular patterns (DAMPs) [30], where injured renal cells may actively release GAS5-containing exosomes into circulation while limiting urinary excretion. This uncertainty, however, provides a foundation for future investigations into the biological pathways governing GAS5’s dual regulatory roles.

The kidney, serving as the principal organ for urine excretion, renders urine composition a revealing “window” into renal pathology. Examining the constituents of urine may offer a promising strategy for uncovering biomarkers linked to renal fibrosis and the progression of CKD [31,32]. Compared to blood samples, urine is more straightforward to collect and encompasses exfoliated cells and secreted substances from diverse intrinsic renal cells, rendering it an optimal source of noninvasive biological data [33,34]. Our study findings revealed that urinary GAS5 levels exhibited a negative correlation with the presence and severity of renal fibrosis. ROC analysis demonstrated that urinary GAS5 outperformed eGFR and TGF-β1 in diagnostic accuracy, with higher AUC values, suggesting urinary GAS5 may serve as a potential biomarker for renal fibrosis. Notably, urinary GAS5 maintained high diagnostic efficacy across fibrosis subgroups, achieving the highest AUC in the severe fibrosis group (*n* = 16), despite its limited sample size, further supporting its diagnostic capability for renal fibrosis. More importantly, its noninvasive nature positions urinary GAS5 as a complementary tool to renal biopsy: ① For high-risk patients with contraindications to biopsy, GAS5 quantification enables dynamic fibrosis monitoring; ② In therapeutic trials, serial measurements could reduce protocol-mandated biopsies while tracking treatment response.

Crucially, when benchmarked against established renal biomarkers, urinary GAS5 exhibits dual advantages: (1) Compared to injury markers (e.g. KIM-1) that peak transiently post-acute damage [35], GAS5 stably tracks chronic fibrotic progression with sustained suppression patterns; (2) Unlike structural proteins (e.g. urinary EGF reflecting tubular reserve) that correlate indirectly with fibrosis [36], GAS5 directly regulates profibrotic miRNA networks [16], providing mechanistically-grounded diagnostic specificity. While urinary exosomal circRNAs [32] show promise, their analytical complexity currently precludes routine clinical adoption, positioning standardized qPCR-based GAS5 quantification as a pragmatically superior approach.

Urinary creatinine (uCr) was selected as the internal reference for GAS5 normalization due to its stable excretion kinetics and physiological relevance to renal filtration efficiency [37]. This approach aligns with clinical standards such as the urine albumin-to-creatinine ratio (UACR) and urine protein-to-creatinine ratio (UPCR), where creatinine corrects for hydration variability and urine concentration fluctuations. However, using random urine samples introduces inherent limitations [37]. Urine sample variability—particularly fluctuations in hydration status and diurnal rhythms—can affect biomarker quantification, as urinary concentrations may vary significantly within individuals across different collection times or hydration conditions [38]. While 24-h urine collection could mitigate these issues, practical challenges such as patient compliance and logistical complexity necessitated the use of random sampling, a common tradeoff in clinical biomarker studies.

While our study provides novel insights into urinary GAS5 as a potential biomarker for renal fibrosis, several limitations warrant consideration. First, the single-center design may limit the generalizability of findings to diverse populations with varying genetic and environmental backgrounds. Second, the cross-sectional nature of data collection precludes establishment of temporal relationships between GAS5 dynamics and fibrosis progression, necessitating future longitudinal studies. Third, inclusion criteria restricted to biopsy-confirmed CKD patients introduces selection bias, potentially excluding early-stage fibrosis cases managed without invasive procedures; multi-center cohorts with broader inclusion criteria are warranted to validate these findings. Fourth, urinary creatinine normalization may introduce measurement variability in severe CKD populations where markedly reduced creatinine excretion could systematically bias GAS5 quantification. Finally, unmeasured confounders such as comorbidities (e.g. heart failure) and medication effects were not accounted for, which may influence GAS5 expression patterns. These limitations highlight opportunities for refining fibrosis biomarker research through multi-center, prospective designs integrating clinical heterogeneity.
